# Dynamic and Polarized Muscle Cell Behaviors Accompany Tail Morphogenesis in the Ascidian *Ciona intestinalis*

**DOI:** 10.1371/journal.pone.0000714

**Published:** 2007-08-08

**Authors:** Yale J. Passamaneck, Anna-Katerina Hadjantonakis, Anna Di Gregorio

**Affiliations:** 1 Department of Cell and Developmental Biology, Weill Medical College of Cornell University, New York, New York, United States of America; 2 Developmental Biology Program, Sloan-Kettering Institute, New York, New York, United States of America; Ecole Normale Superieure, France

## Abstract

**Background:**

Axial elongation is a key morphogenetic process that serves to shape developing organisms. Tail extension in the ascidian larva represents a striking example of this process, wherein paraxially positioned muscle cells undergo elongation and differentiation independent of the segmentation process that characterizes the formation of paraxial mesoderm in vertebrates. Investigating the cell behaviors underlying the morphogenesis of muscle in ascidians may therefore reveal the evolutionarily conserved mechanisms operating during this process.

**Methodology/Principle Findings:**

A live cell imaging approach utilizing subcellularly-localized fluorescent proteins was employed to investigate muscle cell behaviors during tail extension in the ascidian *Ciona intestinalis*. Changes in the position and morphology of individual muscle cells were analyzed *in vivo* in wild type embryos undergoing tail extension and in embryos in which muscle development was perturbed. Muscle cells were observed to undergo elongation in the absence of positional reorganization. Furthermore, high-speed high-resolution live imaging revealed that the onset and progression of tail extension were characterized by the presence of dynamic and polarized actin-based protrusive activity at the plasma membrane of individual muscle cells.

**Conclusions/Significance:**

Our results demonstrate that in the *Ciona* muscle, tissue elongation resulted from gradual and coordinated changes in cell geometry and not from changes in cell topology. Proper formation of muscle cells was found to be necessary not only for muscle tissue elongation, but also more generally for completion of tail extension. Based upon the characterized dynamic changes in cell morphology and plasma membrane protrusive activity, a three-phase model is proposed to describe the cell behavior operating during muscle morphogenesis in the ascidian embryo.

## Introduction

Tissue elongation is a fundamental morphogenetic engine of metazoan development and usually requires dynamic changes in cell topology (the number of contacts with neighboring cells) [1] and/or cell geometry (axial dimensions) along one or more axes. Fundamental to this process are the dynamic cell behaviors and intercellular interactions that act to produce global patterns of tissue rearrangement. Emerging strategies to resolve the behavior of individual cells *in vivo* now provide the potential for unprecedented detail in analyses of morphogenesis.

Due to its rapid development, relative transparency and characteristic features of the chordate body plan, the ascidian *Ciona intestinalis* represents a useful model for embryological studies [2], [3]. Six different main tissues are identifiable in developing *Ciona* embryos and include the muscle, notochord, mesenchyme, endoderm, neural tissue, and epidermis [4]. Of these, muscle, notochord and mesenchyme constitute the three different mesodermal lineages. Like most other cell lineages in the ascidian embryo, the muscle is derived from a fixed number of precursors that produce an invariant final number of differentiated cells (36 muscle cells in the fully developed *Ciona* larva) [4]. Synchronous cell divisions on each side of the ascidian embryo lead to the formation of the final number of muscle cells in the tail by the early tailbud stage (∼9 hours after fertilization at 18°C) [5]. Nonetheless, the tail keeps extending steadily after this stage, for at least an additional 3 hours until the point of larval hatching, by which time it has increased in length more than four-fold.

From an evolutionary perspective, the ascidian muscle lineage may provide a simplified model for analyzing the formation of paraxial mesoderm in chordates. The muscle cells of the ascidian larva are positioned in a paraxial location, where they flank the axially positioned notochord. Ascidian muscle cells express homologs of vertebrate transcriptional regulators required for specification and differentiation of paraxial mesoderm, including *Snail*, *Tbx6* and *MyoD* ([6]; reviewed in [7]). However, unlike the paraxial mesoderm of vertebrates, the ascidian muscle lineage does not undergo segmentation along its anteroposterior axis. Thus, ascidians may provide a system for *in vivo* analysis of the individual cell behaviors underlying paraxial mesoderm formation and differentiation, uncoupled from the segmentation processes of somitogenesis.

To obtain an understanding of the molecular and cellular basis for the morphogenesis and patterning of an embryo, a detailed characterization of the geometry and spatial arrangement of individual cells within developing tissues is required. To this end, we have used *in vivo* imaging to investigate and quantify the baseline muscle cell behaviors taking place in wild type *Ciona* embryos and to contrast these with the cell behaviors operating in a developmentally perturbed context.

We have used live imaging to analyze the behavior of muscle cells *in vivo* in relation to the embryonic context in which they operate, and the global morphogenetic changes they collectively produce. We found that carefully orchestrated and dynamic changes in muscle cell geometry and plasma membrane protrusive activity characterize tail extension in the *Ciona* embryo.

## Materials and Methods

### Ascidians

Adult *Ciona intestinalis* were purchased from Marine Research and Educational Products (M-REP, Carlsbad CA). The animals were kept at 17°C in recirculating artificial seawater. *In vitro* fertilization, dechorionation, and culture of embryos were carried out as described previously [8].

### Plasmid construction

The *sna*>*H2B*-*GFP* vector was constructed by amplifying the human histone *H2B*-*EGFP* fusion gene from the *pCX*::*H2B*-*EGFP* vector [9] using the primers H2B-F (5′-AAT TGC GGC CGC GAT GCC AGA GCC AGC GAA GTC TG-3′) and GFP-R (5′-CAG GTG GCT CAG CTT ACT TGT ACA GCT CGT CC-3′). This fragment was cloned into the *Not*I and *Blp*I sites of the *Ci*-*Bra*-*GFP* vector [8], and the 737 bp *Ci*-*sna* promoter [10] was then cloned into the resultant plasmid at the *Xho*I and *Not*I sites. *sna*>*H2B*-*RFP* was generated by cloning the *Ci*-*sna* promoter into the *Xho*I and *Not*I sites of *Bra*>*mRFP* [11] to generate the *sna*>*RFP* vector, and by subsequently amplifying *H2B* from the *pCX*::*H2B*-*EGFP* vector using the primers H2B-F and H2B-R (5′-ATT CGC GGC CGC CTT AGC GCT GGT GTA CTT GGT G-3′), and cloning the resulting PCR fragment into the *Not*I site of the *sna*>*RFP* vector. The *sna*>*GPI*-*GFP* vector was constructed by amplifying the *GPI*-*EGFP* fusion protein coding sequence from *pCX*::*GPI*-*EGFP* [12], [13] using the primers GPI-EGFP-F (5′-CCT TGC GGC CGC GAT GGT AGA GAT GCT GCC AAC TG-3′) and GPI-EGFP-R (5′-CAC GGT GGC TCA GCT ACA GAG AAA TGA AGT CCA GGG C-3′), and by cloning the resultant fragment into the *Not*I and *Blp*I sites of *Bra*>*EGFP*, followed by insertion of the 737 bp *Ci*-*sna* promoter at the *Eco0109*I and the *Not*I sites. *Tbx6b*>*H2B*-*GFP* was generated by cloning an *Xho*I/*Not*I fragment from *Tbx6b*>*lacZ*, which contains a 1.5 kb muscle enhancer of *Ci*-*Tbx6b* (J. Kugler and A.D.G., unpublished results), into the *Xho*I and *Not*I sites of *sna*>*H2B*-*GFP*. *Tbx6b*>*GPI*-*GFP* was generated by cloning the *Xho*I/*Not*I fragment from *Tbx6b*>*lacZ* into the *Xho*I and *Not*I sites of *sna*>*GPI*-*GFP*. *Tbx6b*>*PH*-*YFP* was generated by cloning a fusion of the PLC delta pleckstrin homology (PH) domain and *YFP*, derived from the *PH*-*EGFP* construct [14] and a *Ciona* codon optimized version of *YFP* [15] into the *Not*I and *Blp*I sites of *Tbx6b*>*GPI*-*GFP*.

### Electroporations

Purified circular plasmid DNA was prepared using the NucleoBond plasmid kit (BD Biosciences, San Jose CA) and used to electroporate one-cell *Ciona* embryos as described previously [8]. Each transgene was tested in at least three independent experiments using different batches of embryos.

### Vital dye staining

Embryos were incubated in FM4-64 (Molecular Probes, Carlsbad CA) at a final concentration of 20 µM in sea water for 1 hour prior to imaging.

### Cytochalasin D treatment

Embryos were grown to the neurula stage and transferred into a solution of 500 nM cytochalasin D (Calbiochem, San Diego CA) in filtered seawater. Embryos were incubated in cytochalasin D for 15 minutes prior to imaging. For measurements of tail extension, embryos were treated with cytochalasin D for one hour, and were then recovered into filtered seawater and maintained until untreated control embryos from the same batch had reached the late tailbud stage.

### Phalloidin staining

Embryos were grown to the swimming larval stage and fixed in 4% formaldehyde in seawater for 30 minutes. After three washes in PBT, larvae were incubated with rhodamine phalloidin (Molecular Probes, Carlsbad CA) at a concentration of 5 U/ml in PBT for 30 minutes. Following labeling, the larvae were washed three times in PBS prior to mounting for imaging.

### Transmission electron microscopy

Swimming control larvae and larvae electroporated with *sna*>*Bix* were fixed by suspension and immediate pelleting in 2.5% glutaraldehyde in 0.1 M sodium cacodylate buffer that contained 0.34 M NaCl and incubated overnight at 4°C. After three washes in 0.1 M sodium cacodylate buffer, larvae were post-fixed for 1 hr. in 1% osmium tetroxide and 1.5% potassium ferricyanide (aqueous), washed as above, rinsed in deionized water and en-bloc stained with aqueous 3% uranyl acetate for 1 hr. After dehydration through an ethanol series, the samples were infiltrated with increasing concentrations of Spurr's resin and left at 60°C for ∼24 hr.

Semi-thin (0.5 um) and ultra-thin (65 nm) sections were cut using a Diatome diamond knife on a Leica Ultracut S ultramicrotome. Ultra-thin sections were further contrasted with lead citrate, then viewed in a JEOL JEM 100CX-II electron microscope. Images were recorded on Kodak 4489 electron microscope film. Negatives were scanned into digital format.

### *In vivo* image acquisition

All confocal images shown are of living embryos maintained under physiological conditions. Embryos were viewed for onset of transgene activity and for determination of electroporation efficiencies under a Leica MZFLIII stereo dissecting microscope equipped with epifluorescence illumination and appropriate filters for visualizing fluorophores of interest. Embryos were mounted on coverslip-bottomed dishes (MatTek, Ashland MA) for confocal imaging. Laser scanning confocal data was acquired with a LSM510 META (Zeiss, Thornwood NY) on a Zeiss Axiovert 200M. Spinning disc confocal data were acquired with an Ultra View spinning disc confocal (Perkin Elmer, Waltham MA) on a Zeiss Axiovert 200M. *z*-stacks were taken at 0.3–2.0 µm intervals at time intervals of 30–300 seconds. For each experiment, several hundred electroporated embryos were obtained. One representative embryo was selected for imaging, while the remaining embryos were maintained as stage-matched controls.

### Image processing

Raw data were processed using Imaris (Bitplane AG at http://www.bitplane.com/), Volocity (Improvision at http://www.improvision.com/) or Image J (http://rsb.info.nih.gov/ij/) software. Each image series was re-animated and/or annotated using Adobe Premiere Pro software (Adobe at http://www.adobe.com/) or QuickTime Player (Apple Computer, Inc. at http://www.apple.com/quicktime/).

### Image quantitation

Analysis of cell positions was conducted by manually selecting nuclei using the Volocity software and by measuring centroid positions. Nuclear positions and internuclear distances were plotted using Microsoft Excel (Microsoft at http://www.microsoft.com). To normalize the data for movements of the embryo within the field of view, which occurred during the course of imaging, the anterior tip of the embryo's trunk was considered to be a fixed point in space (origin), and all measurements were corrected accordingly. Embryos also rotated about the *z*-axis as the tail extended, complicating comparisons between time points. To correct for this rotation, the line extending from the anterior tip of the trunk to the ventral notch at the intersection of the trunk and the tail was considered to have an angle ø = 0° at all time-points. Tailbud embryos were staged based on tail length (measured as the distance along the midline from the anterior tip of the notochord to the posterior tip of the tail), due to the fact that the rate of development in ascidians varies according to the ambient temperature [5].

## Results

### Elongation of muscle tissue during tail extension

**Figure 1:**
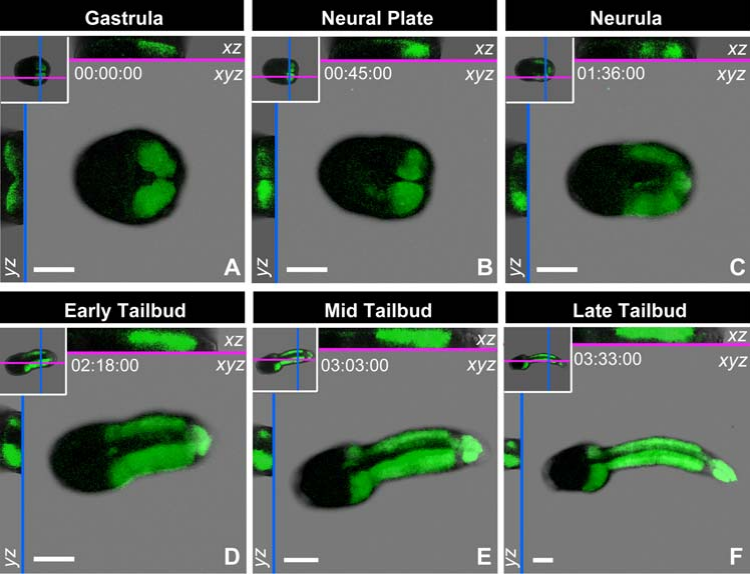
3D time-lapse series of muscle development in a *Ciona intestinalis* embryo visualized by electroporation with the *sna*>*Venus* plasmid. (A) gastrula, (B) neural plate, (C) neurula, (D) early tailbud, (E) mid tailbud, and (F) late tailbud stages from a single embryo are shown. The anterior of the embryo is to the left, posterior to the right. A maximum intensity projection of all slices along the *z*-axis is shown in the *xy*-plane view for each stage. Cross-sectional slices in the *xz* and *yz*-planes are shown above and to the left of the *xy*-axis view, respectively, and the positions of the slices are represented in the insets. Time stamps are shown in each panel. Scale bars represent 40 µm.

To selectively visualize muscle cells we used a promoter isolated from the *Ciona intestinalis snail* locus [10]. The *sna*>*Venus* reporter construct, driving expression of the yellow fluorescent protein Venus [16], allowed us to label and follow the muscle lineage *in vivo* in developing *Ciona* embryos with no deleterious effects on development (Fig. 1; Movie S1) [11]. *In vivo* imaging of fluorescently labeled muscle cells in *sna*>*Venus* transgenic animals highlighted the elongation of the muscle tissue during the course of tail extension (Fig. 1; Movie S1). Fluorescence was first detected at gastrulation (Figure 1A); thereafter, embryos were 3D time-lapse (i.e. 4D) imaged until the late tailbud stage, without perturbing normal development. During gastrulation, the muscle precursors underwent invagination (Figure 1A,B), and became positioned bilaterally during the neurula stage (Figure 1C). From the early to late tailbud stages the muscle lineage was seen to exhibit a considerable elongation, increasing in length more than four-fold along the anteroposterior axis as the embryo underwent tail extension (Figure 1D–F).

### Tail extension in the absence of muscle cell division or intercalation

Although live imaging of *sna*>*Venus* transgenic embryos offered an unprecedented visualization of muscle tissue morphogenesis throughout the embryonic development of *Ciona*, the widespread cytoplasmic distribution of the native Venus fluorescent protein did not permit resolution of individual muscle cells (cross-sectional slices in Figure 1). Since labeling different sub-cellular compartments currently provides the highest resolution read-out of *in vivo* cell behavior and cell fate [17]–[19], we went on to generate and 3D time-lapse image transgenic animals expressing spectrally distinct, subcellularly-localized fluorescent proteins to label the nuclei and plasma membranes of muscle cells.

**Figure 2:**
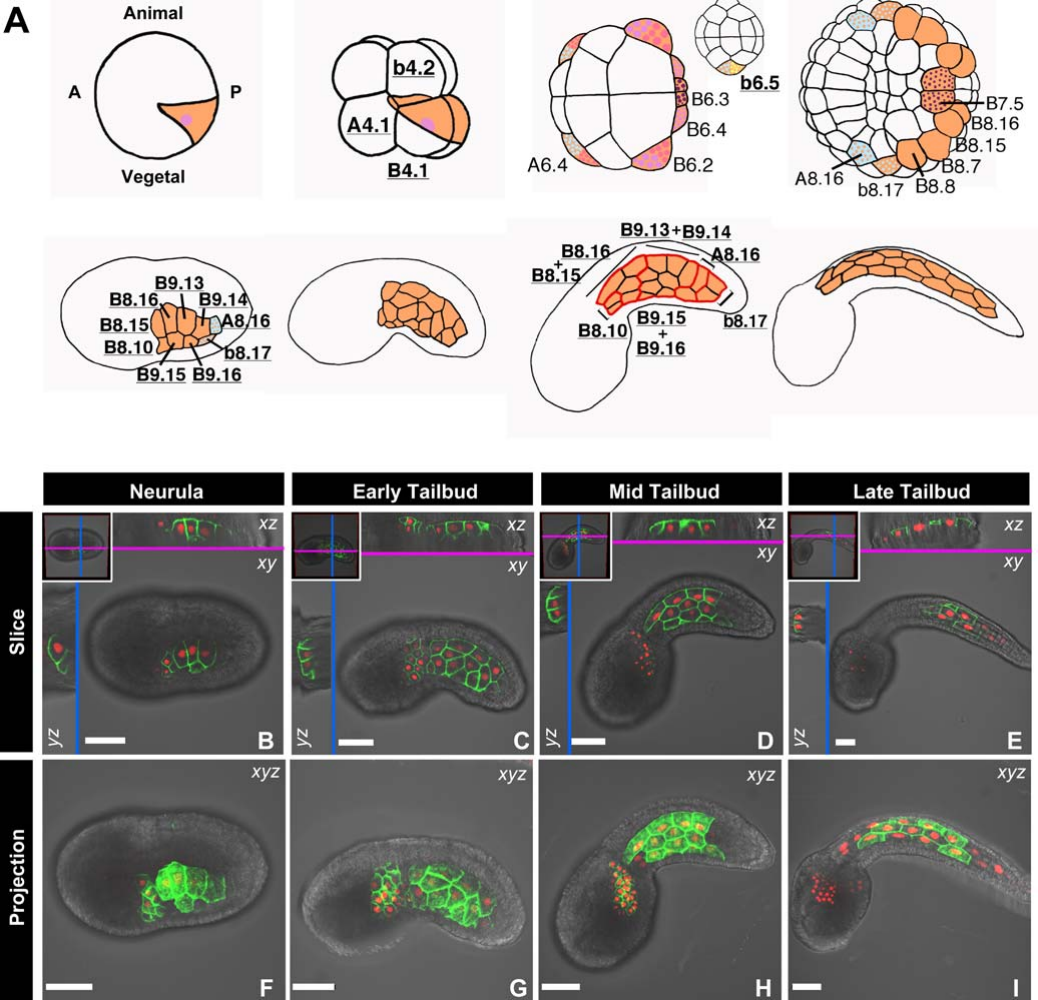
Schematic illustrations depicting muscle cell development in *Ciona* and high-resolution imaging of muscle development by dual-tagging muscle cells with spectrally distinct, subcellularly-localized fluorescent proteins. (A) Schematics of the muscle lineage of the *Ciona intestinalis* embryo at the one-cell, 8-cell, 32-cell, 110-cell, neurula, early tailbud, mid tailbud and late tailbud stages, with the cell lineages marked by conventional nomenclature. Only one side of the embryo is labeled. Tail muscle precursors are labeled in orange, neural tissue in light blue, trunk mesenchyme in light purple and trunk ventral cells (heart progenitors) in dark purple. Blastomeres that give rise to more than one tissue are stippled with the colors corresponding to their fates. (B–I) Time series of embryos co-electroporated with *sna*>*GPI*-*GFP* and *sna*>*H2B*-*RFP*. (B,F) neurula, (C,G) early tailbud, (D,H) mid tailbud, and (E,I) late tailbud stages are shown. (B–E) A single slice in the *z*-axis is shown in the *xy*-plane of view. Cross-sectional slices in the *xz* and *yz*-planes are shown above and to the left of the *xy*-axis view, respectively, and the positions of the slices are represented in the insets. (F–I) A maximum intensity projection of all slices along the *z*-axis is shown in the *xy*-plane of view. Scale bars, 40 µm.

The invariant cell lineage of the *Ciona* muscle provides the potential for analyzing morphogenesis at the scale of individual cell behaviors (Figure 2A). To visualize the position of individual cells within the muscle tissue and assess their role in tail extension, we labeled their nuclei with an H2B-RFP reporter (Figure 2B–I; Movie S2) comprising the monomeric red fluorescent protein mRFP1 fused to human histone H2B, which labels active chromatin [9]. In embryos electroporated with *sna*>*H2B*-*RFP*, chromatin localized red fluorescence was first detected in muscle precursor cells at the neurula stage (Figure 2B) and persisted through the course of tail extension (Figure 2C–E). In agreement with previous data generated from bright field visualization of ascidian embryos [20], muscle cells were observed to undergo their final cell division at the neurula stage, with no proliferation occurring during the period of tail extension.

To analyze changes in the morphology of individual muscle cells during tail extension we labeled the cell membranes with a plasma membrane marker to allow identification of cell boundaries and therefore provide information on cell morphology and topology [1]. Muscle cell boundaries were specifically labeled by a GFP fusion protein containing a glycosylphosphatidylinositol (GPI) tag, which was encoded by the *sna*>*GPI*-*GFP* plasmid (Figure 2B–I, Movie S2) [13].

Plasma membrane localized green fluorescence was first detected in muscle precursors at the gastrula stage in embryos electroporated with *sna*>*GPI*-*GFP* (data not shown). Prior to tail extension, during the neurula stage, muscle cells became positioned paraxially to the notochord (Figure 2B). During the early tailbud stage (Figure 2C) they underwent only minor refinements in their position relative to their neighbors before adopting a final and invariant position that was retained throughout the mid and late tailbud stages (Figure 2D,E; Movie S2). In this final configuration, muscle cells were aligned on either side of the notochord, as three rows of cells along the anterior two thirds of the length of the tail and as two rows at the distal end of the tail (Figure 2I). This final arrangement of muscle cells is highly stereotyped and was observed in all wild type embryos (data not shown).

Once their position and nearest neighbor contacts were locked in their terminal position, cells in the medial row of the muscle exhibited hexagonal topologies characteristic of epithelial cells (Figure 2I) [1]. The adjacent dorsal and ventral rows of muscle cells, which provide the interface with other tissues, also had straight epithelial-like borders. However, these cells were usually pentagonal, with those borders that interfaced with other tissues aligned perpendicularly to the dorsoventral axis (Figure 2I).

During tail extension, individual muscle cells did not undergo extensive rearrangements in their relative positions (Figure 2G–I); however, the continuous epithelial sheet they formed on either side of the notochord underwent a ∼4-fold elongation during tail extension. Together, these observations suggest that changes in cell geometry (length versus height) must play a major role in this process.

Consistent with our results from electroporation of *sna*>*Venus*, both *sna*>*H2B*-*RFP* and *sna*>*GPI*-*GFP* were found to be developmentally neutral, even when co-electroporated to simultaneously label and image two compartments of the same cell (Figure 2I). The settings for image acquisition were optimized so that in all experiments expression of fluorescent proteins and confocal imaging were found to have no effect on embryos. Both the onset of otolith melanization and muscle contractions were synchronous between imaged and control embryos (Figure S1).

### Muscle cell expansion during tail extension

**Figure 3:**
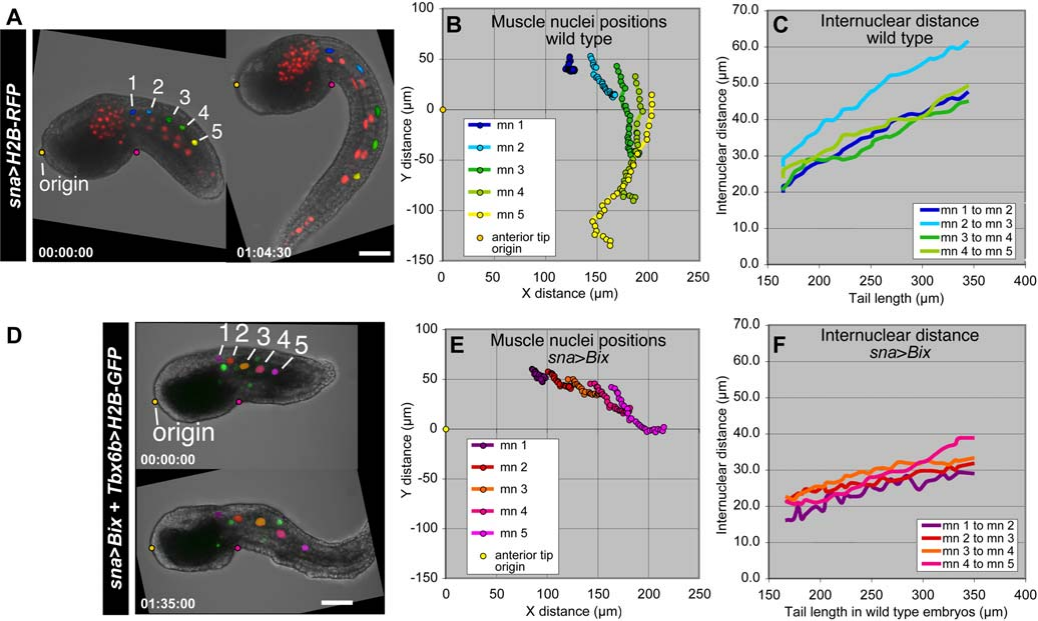
Tracking the position of muscle nuclei during tail extension in wild type and perturbed embryos. In each case, the same embryo is shown at the early tailbud (left) and late tailbud (right) stages. Nuclei followed for tracking are highlighted with false colors and numbered. The anterior tip of the embryos (origin) and the notch at the intersection of the trunk and the tail, used to correct for movement in the frame, are highlighted in yellow and pink, respectively. Time stamps are shown in each panel. (A) A wild type embryo electroporated with *sna*>*H2B*-*RFP*, and (D) a perturbed embryo co-electroporated with *sna*>*Bix* and *Tbx6b*>*H2B*-*GFP*. (B, E) Paths of movement of selected muscle cell nuclei (mn), relative to the position of the anterior tip, in wild type (B) and *sna*>*Bix* (E) embryos. (C, F) Change in internuclear distance between nearest neighbor muscle cells during tail extension in unperturbed (C) and *sna*>*Bix* (F) embryos. Tail lengths for the *sna*>*Bix* (F) embryo were measured in parallel from a stage-matched wild type embryo. Because *sna*>*Bix* affects tail extension, staging of *sna*>*Bix* embryos was based upon measurements of wild type embryos cultured in parallel from the same batch. Wild type embryos were monitored on a wide-field inverted microscope located directly adjacent to the laser scanning confocal microscope, to ensure that the two samples were maintained in similar environmental conditions. Scale bars, 40 µm.

Given that elongation of the muscle tissue is achieved in the absence of cell proliferation, it may be expected that expansion is coordinated and takes place along the entire length of the tail, at a constant or variable rate, rather than occurring only at a posteriorly positioned growth zone. To measure the movements of individual cells positioned at different points along the anteroposterior length of the tail we used *sna*>*H2B*-*RFP* to track the positions of muscle cell nuclei over the course of tail extension (Figure 3A; Movie S3). The positions of individual muscle cell nuclei were tracked from the early tailbud to the late tailbud stage (1 hour, 30 minutes; Figure 3A,B). To plot the movement of multiple nuclei over time the anterior tip of the embryo was considered to remain fixed in space at the origin for all time points (Figure 3B). During tail extension muscle cells showed an overall movement in the *y*-axis relative to the position of the origin, likely accounting for the increase in tissue length (Figure 3B). The more posterior cells also displayed a vector component trending towards the origin along the *x*-axis, caused by the curling of the tail during its extension. Late in tail extension these posterior nuclei moved away from the origin, as the tail began to uncurl (Figure 3B).

Throughout the course of tail extension the distance between nearest-neighbor pairs along the anteroposterior axis increased in a linear fashion, and the rate of expansion was similar for all pairs (Figure 3C). Although the distance between “mn 2” and “mn 3” was greater than that between other pairs of cells, the rate of expansion was equivalent to that of other pairs. These results demonstrated that tail extension was accompanied by an expansion of muscle cells along the length of the tail, resulting in a constant increase in distance between all pairs of muscle nuclei along the anteroposterior axis.

To analyze muscle cell development in a perturbed developmental context we misexpressed the *Xenopus* homeodomain-containing transcription factor Bix1 [21] in muscle precursors, using the *sna*>*Bix* construct, which had previously been shown to cause shortening and/or bending of the tail when electroporated in *Ciona* embryos [22]. Although *Ciona* mutants characterized by an impairment in tail extension have been previously described, such mutations are attributable to specific defects in notochord development [23], [24]. Since no ascidian mutants with specific perturbations in muscle morphogenesis have yet been described, *sna*>*Bix* represented the best available tool for perturbing this process without impeding either specification of muscle cells or the initial stages of their differentiation.

From the mid tailbud to the late tailbud stages, embryos electroporated with *sna*>*Bix* displayed a rate of tail extension that was approximately 50% that of wild type embryos from the same batch cultured in parallel (Figure S2B; 1.2 µm per minute for *sna*>*Bix* embryos versus 2.4 µm per minute for the wild type embryo). Although tail extension was impaired in embryos electroporated with *sna*>*Bix*, muscle cell specification and differentiation were observed. For example, expression levels of three markers of muscle differentiation, *muscle actin*, *myosin regulatory light chain*, and *troponin I*, were found to be similar in wild type and *sna*>*Bix* embryos when assayed by *in situ* hybridization (Figure S3). However, *sna*>*Bix* embryos showed a delay in the onset of muscle contractions as compared to wild type embryos (Figure S4, Movie S4), while fully developed *sna*>*Bix* larvae were unable to perform the coordinated muscle contractions necessary for swimming (data not shown). Labeling of F-actin with rhodamine phalloidin suggested that muscle cells expressing Bix1 have fewer, less organized myofibrils than do muscle cells in wild type embryos (Figure S5). These data were confirmed by transmission electron microscopy (Figure S6).

To investigate differences in muscle morphogenesis between wild type and developmentally perturbed embryos, we utilized the labeling tools and applied the metrics described previously to analyze embryos electroporated with the *sna*>*Bix* construct. The fluorescent reporter plasmids containing the *Ci*-*sna* promoter were found to be unsuitable markers for these experiments due to a reduction of fluorescence in the presence of Bix1, likely caused by a downregulation of the *sna* promoter element. To overcome this limitation, we analyzed the *sna*>*Bix* phenotype using another muscle enhancer, isolated from the *Ciona Tbx6b* gene (J. Kugler and A.D.G, unpublished results). Measurements of wild type embryos electroporated with the *Tbx6b* constructs were equivalent to those of embryos bearing *sna* transgenes (data not shown). Unlike *sna*>*H2B*-*GFP* or *sna*>*H2B*-*RFP*, the activity of *Tbx6b*>*H2B*-*GFP* was not reduced in embryos co-electroporated with *sna*>*Bix*. Therefore, characterization of the muscle phenotype induced by Bix1 was conducted using embryos co-electroporated with *sna*>*Bix* and *Tbx6b*>*H2B*-*GFP* (Figure 3D; Movie S5).

Comparisons made between muscle cells in similar positions in the tails of wild type and *sna*>*Bix* embryos demonstrated that muscle nuclei in perturbed embryos underwent less movement than did comparable cells in control embryos (Figure 3B,E). The overall direction of movement of *sna*>*Bix* muscle nuclei was away from the anterior tip; however, the curvature of movement tracks that is characteristic of wild type embryos was not observed in *sna*>*Bix* embryos (Figure 3E). Muscle nuclei in *sna*>*Bix* embryos did exhibit an increase in internuclear distance during tail extension, but the rate of change in internuclear distance was considerably reduced compared to that of wild type embryos (Figure 3F; 0.06 in the *sna*>*Bix* embryo versus 0.16 in the wild type embryo; values are unitless because they represent ratios of internuclear distance over tail length).

### Muscle elongation during tail extension is accomplished through changes in cell geometry

**Figure 4:**
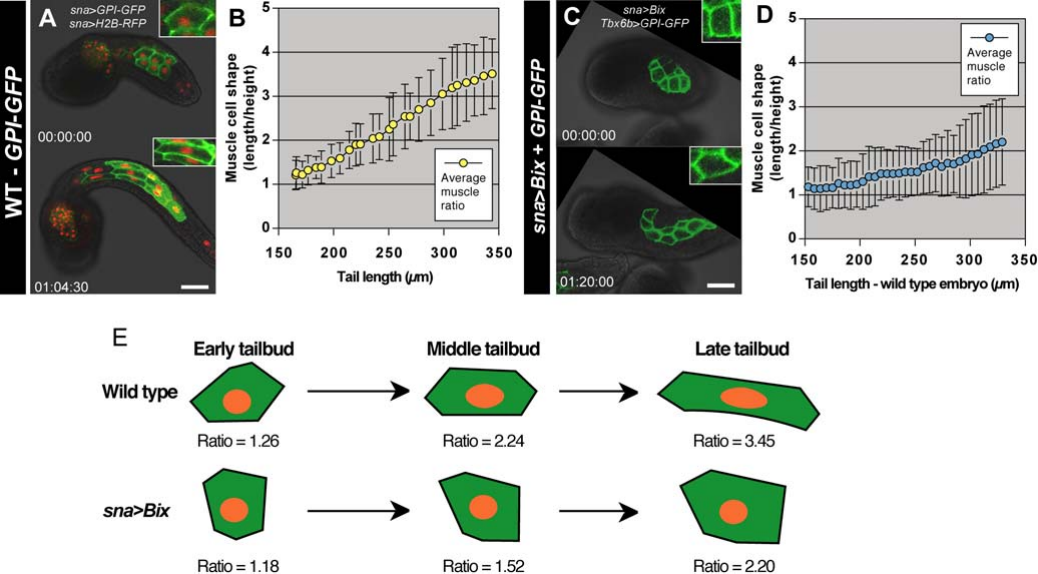
Changes in muscle cell geometry during tail extension. (A) Muscle cell boundaries highlighted by GPI-GFP in an embryo co-electroporated with *sna*>*GPI*-*GFP* and *sna*>*H2B*-*RFP*, shown at early tailbud and late tailbud stages. (B) Average muscle cell shape (ratio of length to height; n = 5) from the *sna*>*GPI*-*GFP* + *sna*>*H2B*-*RFP* embryo plotted against tail length, with standard deviation shown for each data point. (C) Embryo co-electroporated with *sna*>*Bix* to perturb muscle development and *Tbx6b*>*GPI*-*GFP* to mark muscle membranes. (D) Graph of average muscle cell shape (ratio of length to height; n = 5) from the *sna*>*Bix* + *Tbx6b*>*GPI*-*GFP* embryo plotted against tail length of a wild type embryo monitored in parallel, with standard deviation shown for each data point. (E) Schematic of muscle cell shape changes in wild type and *sna*>*Bix* embryos during tail extension. Time stamps are shown in each image panel. Scale bars, 40 µm.

To analyze the role of cell morphology dynamics in the process of muscle tissue elongation, we used the *sna*>*GPI*-*GFP* plasmid to visualize muscle cell topology and to quantify muscle cell geometry in tailbud stage embryos (Figure 4A; Movie S6). Cell geometry was calculated as the ratio of cell length (medial length of the cell parallel to the anteroposterior axis of the embryo) to cell height (medial length of the cell perpendicular to the anteroposterior axis of the embryo). The average ratio of length to height in five cells imaged within a single embryo was found to be close to 1∶1 at the early tailbud (tail length = 160 µm; Figure 4B). This ratio increased steadily over the course of tail extension up to a ratio of 3.5∶1 at the late tailbud stage (tail length = 350 µm; Figure 4B). These numbers correlated with similar observations made in four other embryos (data not shown).

To further understand the role of muscle elongation in tail extension, we measured the changes in muscle cell shape in *sna*>*Bix* embryos co-electroporated *sna*>*Bix* with the *Tbx6b*>*GPI*-*GFP* construct to label cell boundaries (Figure 4C; Movie S7[Supplementary-material pone.0000714.s014]). The majority of muscle cells labeled in these perturbed embryos did not take on the stereotypic hexagonal and pentagonal topologies that characterized the ordered arrangement of muscle cells in wild type embryos. Muscle cells in *sna*>*Bix* embryos displayed a disordered arrangement, with most cells having trapezoidal or pentagonal topologies (Figure 4C). Rates of muscle cell elongation in *sna*>*Bix* embryos were much lower than those in wild type embryos (Figure 4D).

To confirm these results using an analogous labeling technique, muscle cell shape changes were also measured in embryos incubated in the vital dye FM4-64, which labels cell membranes. In embryos incubated in FM4-64, fluorescence was observed specifically in cell membranes and development was not perturbed (Figure S). Muscle cells labeled with FM4-64 showed comparable rates of elongation to those labeled with GPI-GFP (Figure S7A,B). However, FM4-64 was more susceptible to photobleaching than was GPI-GFP, and was internalized by the cells over time, therefore permitting only short-term plasma membrane labeling and live imaging (data not shown). Since FM4-64 labeling lacked tissue specificity, embryos were electroporated with *Tbx6*>*H2B*-*GFP* to allow identification of muscle cells (Figure S7A).

Muscle geometry was also measured in *sna*>*Bix* embryos co-electroporated with *Tbx6b*>*H2B*-*GFP* and counter-stained with FM4-64 (Figure S7C). Muscle cells in *sna*>*Bix* embryos showed minimal changes in geometry during the period of tail extension (Figure S7D). The differences in the rate of muscle extension between *sna*>*Bix* embryos coexpressing *Tbx6b*>*GPI*-*GFP* (Figure C,D) and those labeled with FM4-64 (Figure S7C,D) likely reflected the variability in the expression levels of Bix1 due to mosaic incorporation of the *sna*>*Bix* plasmid [22].

Nevertheless, muscle cells in *sna*>*Bix* embryos exhibited an average increase in the ratio of length to height of less than 2-fold, while over the same period the ratio increased by nearly 3-fold in wild type embryos (Figure 4E). Overall, the tails of *sna*>*Bix* embryos failed to extend, as compared to wild type embryos (Figure S2B). These results further support the observation that changes in muscle cell geometry underlie muscle elongation in the absence of cell division.

**Figure 5:**
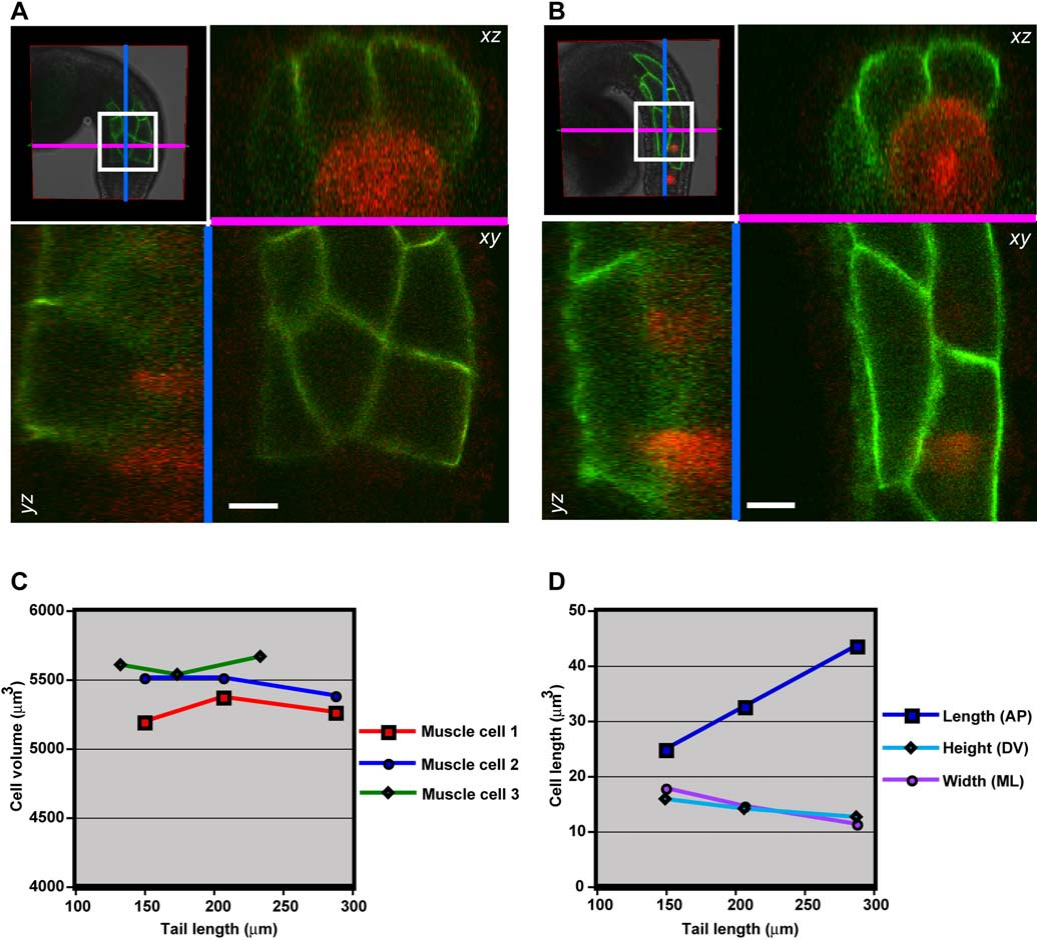
Muscle cell volume during tail extension. (A) Cross-sectional slices of GPI-GFP labeled muscle cells at the early tailbud stage. The underlying notochord cells are highlighted by coelectroporation of *Bra*>*RFP* [11]. (B) Cross-sectional slices of the same muscle and notochord cells at the late tailbud stage. (C) Graph of cell volumes for three muscle cells during tail extension. (D) Graph of changes in anteroposterior length, dorsoventral height and mediolateral width during tail extension in “muscle cell 1” from the previous panel. Scale bars, 10 µm.

Despite the remarkable morphogenesis of the muscle lineage during tail extension, measurements of the 3D time-lapse data revealed that muscle cells undergo no volumetric growth during this process (Figure 5). To evaluate the potential role of muscle cell growth in muscle elongation, muscle cell volume was measured during the course of tail extension in wild type embryos (Figure 5A,B). The volumes of muscle cells were found, on average, to remain constant over the course of tail extension (Figure 5C). This suggests that the process of muscle elongation is accomplished through axial deformation of muscle cells, rather than through volumetric growth. Consistent with this, the increase in anteroposterior length of muscle cells was accompanied by decreases in both their dorsoventral height and mediolateral width (Figure 5D).

### Developmental changes in muscle cell surface projections

**Figure 6:**
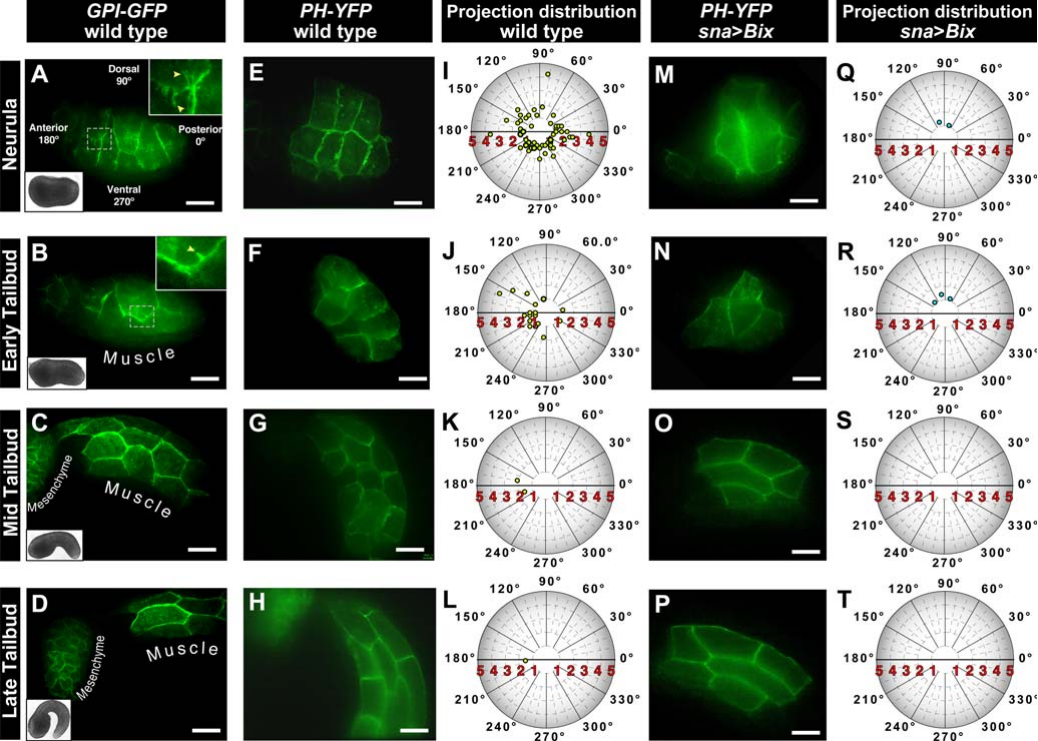
Differential protrusive activity at the plasma membrane of muscle cells during tail extension. (A) Neurula, (B) early tailbud, (C) mid tailbud and (D) late tailbud stage embryos electroporated with *sna*>*GPI*-*GFP* and imaged by high-speed spinning disk confocal microscopy. Brightfield images of stage matched embryos are shown in the bottom left corner of each panel. High magnification views of the muscle cells membranes outlined by dashed lines are shown in panels A and B. (E–H) Actin-based cell surface projections imaged by electroporation of *Tbx6b*>*PH*-*YFP*. (I–L) Polar plots of muscle cell protrusion length and orientation in neurula, early tailbud, mid tailbud, and late tailbud embryos. Protrusion length and angle are shown as the distance from the center of the plot and the angle from the *x*-axis respectively. (M–P) Cell surface projections in perturbed embryos. Embryos were co-electroporated with *sna*>*Bix* and *Tbx6b*>*PH*-*YFP*. (Q–T) Polar plots of muscle cell protrusion length and orientation in perturbed embryos co-electroporated with *sna*>*Bix* and *Tbx6b*>*PH*-*YFP*. Distance in µm is shown along the *x*-axis. Scale bars, 20 µm.

The precise molecular mechanisms underlying the profound changes in cell shape during muscle elongation in the ascidian remain mostly unknown. To assess the role of intercellular interactions in the muscle during tail extension, we performed a detailed study of protrusive activity at the plasma membrane of muscle cells labeled using the *sna*>*GPI*-*GFP* construct. High-speed imaging of *sna*>*GPI*-*GFP* embryos using a spinning disc confocal microscope resolved transient projections extending from the lateral surface of muscle cells (Figure 6A–D and Movie S8). Such projections were not detected by laser scanning confocal microscopy, likely due to the longer acquisition times necessary for each *xy* frame. Projections could first be imaged on the surface of muscle cells at the neurula stage, extending from one muscle cell to another (Figure 6A and Movie S8). Individual projections were transient, with an average duration of less than one minute. The average length of the projections was 1.5 µm from base to tip (n = 43).

To gain insight into the molecular nature of the muscle cell surface projections we employed additional fluorescent protein fusions that demarcate components of the cytoskeleton. F-actin was imaged using a pleckstrin homology (PH) domain fluorescent protein fusion encoded for by the *sna*>*PH*-*YFP* construct. When previously fused to GFP, the phospholipase C-delta 1 PH domain has been shown to associate with, and provide a read out of, F-actin polymerization at the cell surface [14], [25]. Embryos electroporated with *sna*>*PH*-*YFP* displayed plasma membrane protrusive activity that was indistinguishable from that observed in embryos electroporated with *sna*>*GPI*-*GFP*, suggesting that the projections are likely actin-based (Figure 6E–H and Movie S9).

We next quantified the number and orientation of these actin-based plasma membrane projections (Figure 6I–L). Muscle cell surface projections were most abundant at the neurula stage (Figure 6A,I; 2.1±0.8 projections/cell, n = 28), decreased in number by the early tailbud stage (Figure 6B,J; 1.5±0.1 projections/cell, n = 12), and were almost entirely absent by the mid tailbud (Figure 6C,K) and late tailbud stages (Figure 6D,L). No clear trend was observed in the orientation of projections at the neurula stage (Figure 6I). However, by the early tailbud stage the majority of projections were oriented anteriorly (Figure 6J).

### Muscle cell surface projections are reduced in developmentally perturbed embryos

To test whether muscle cell surface projections were affected when muscle elongation was perturbed, embryos were electroporated with *sna*>*Bix* and *Tbx6b*>*PH*-*YFP* (Figure 6M–P and Movie S10). *sna*>*Bix* embryos displayed a reduction in the number of projections at both the neurula (Figure 6M,Q) and early tailbud (Figure 6N,R) stages, as compared with wild type embryos. Projections were also absent from mid tailbud (Figure 6O,S) and late tailbud (Figure 6P,T) stage embryos, suggesting that formation of these projections is impaired in *sna*>*Bix* embryos, rather than being delayed in onset. In *sna*>*Bix* embryos the failure of muscle cells to adopt their proper topology and geometry is correlated with a reduction in the number of plasma membrane projections.

**Figure 7:**
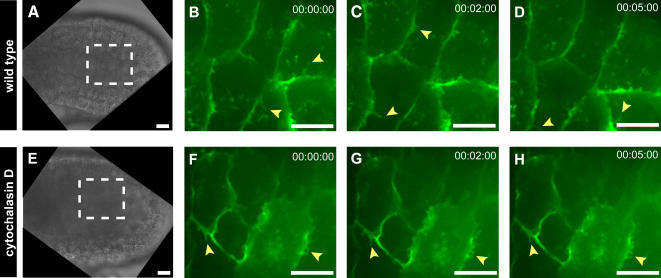
Impairment of muscle cell activity by cytochalasin D. (A) Brightfield image of a wild type neurula stage embryo, with dashed lines outlining the region shown in high magnification in panels B–D. (B–D) High-speed confocal images of a wild type embryo electroporated with *sna*>*GPI*-*GFP*. Yellow arrowheads highlight dynamic projections, which had a duration of less than two minutes. (E) Brightfield image of a neurula stage embryo incubated in 500 nM cytochalasin D, with dashed lines outlining the region shown in high magnification in panels F–H. (F–H) High speed confocal images of an embryo electroporated with *sna*>*GPI*-*GFP* and treated with cytochalasin D. Blue arrowheads highlight projections, which were static throughout the 5 minute imaging period. Scale bars, 10 µm.

To shed light on the morphogenetic role of muscle cells projections, embryos were treated with cytochalasin D, a potent inhibitor of actin polymerization [26]. Embryos incubated in 500 nM cytochalasin D exhibited an arrest in the dynamics of muscle cell projections (Figure 7). While in wild type embryos the average duration of projections was less than 2 minutes (Figure 7A–D), in embryos treated with cytochalasin D the average duration of projections was greater than 5 minutes (Figure 7E–H). Cytochalasin D also affected the process of tail extension, mimicking the effects of *sna*>*Bix* expression. Embryos incubated in cytochalasin D for one hour at the neurula stage, and recovered into filtered seawater, displayed significantly shorter tails at the late tailbud stage than did wild type embryos (214±29 µm (n = 16) for cytochalasin D treated embryos versus 306±30 µm (n = 12) for wild type embryos). Latrunculin B, which similarly impairs actin polymerization, was also tested at different concentrations, but exhibited high levels of cytotoxicity as compared with cytochalasin D (data not shown).

## Discussion

A detailed characterization of the morphogenetic events that constitute normal embryonic development is essential for understanding the regulation of developmental processes and the patterning mechanisms underlying them. By combining 3D time-lapse imaging with transient transgenesis in ascidian embryos we have investigated *in vivo* wild type and perturbed muscle development at the cellular level. *Ciona* embryos, due to their overall size, transparency and rapid development provide an ideal model system for such multidimensional live imaging studies.

### High-resolution imaging of developing muscle cells using spectrally distinct subcellularly-localized fluorescent reporters

Analysis of developing muscle cells was achieved at an unprecedented resolution in living *Ciona* embryos by means of subcellularly-localized fluorescent proteins, which allowed visualization of muscle cell behavior *in situ* during tail extension. Human histone H2B-tagged fluorescent proteins provided markers of cell position and division during the course of development [27], [28], and were used to identify and track individual muscle cell positions in 3D over time. Additionally, lipid-modified GPI-tagged fluorescent proteins highlighted the cell surface, permitting quantitative measurements of cell shape changes during tail extension and visualization of a novel dynamic protrusive activity at the plasma membrane. Furthermore, *in vivo* imaging of a PH-YFP fusion indicated that this protrusive activity was actin-based.

### Stereotypical changes in muscle cell geometry underlie tail extension

We have used live imaging to generate a detailed *in vivo* description of cell behaviors underlying tail extension in the absence of cell division or intercalation. *Ciona* muscle cells were observed to undergo changes in their geometry during tail extension starting at the neurula stage, elongating along the anteroposterior axis (Movie S2). This change in geometry occurred without changes in cell topology, as cells remained locked in their position relative to their neighbors. Morphometric analysis of the imaging data we have collected demonstrates that the rate of elongation is linear and that by the late tailbud stage, cell length (along the anteroposterior axis) exceeds by almost four times the cell height (along the dorsoventral axis). Our analyses of the positions of nuclei demonstrate that the rate of expansion is consistent between muscle cells along the entire length of the tail during tail extension. Elongation of the muscle during tail extension is accomplished entirely through changes in the geometry of individual muscle cells in the absence of intercalation or other changes in the relative positions of cells.

### A three-phase model for cell behavior during muscle morphogenesis

**Figure 8:**
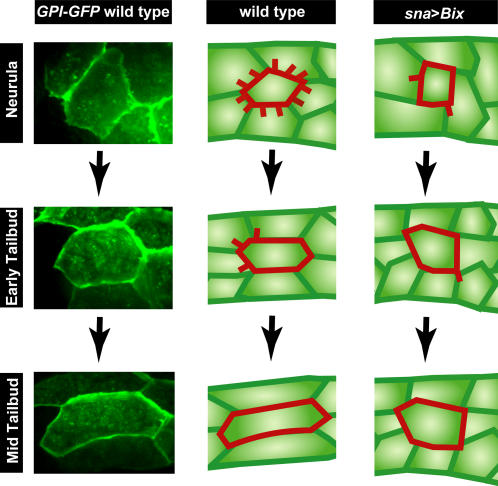
Three-phase model of cell behavior operating during tail extension in *Ciona*. High magnification photomicrographs of muscle cells expressing GPI GFP are shown in the left column. Schematics of wild type muscle cells are shown in the middle column, with the borders and projections of a single cell outlined in red. Schematics of muscle cells expressing Bix1 are shown in the right column. Muscle cells undergo their last division at the neurula stage. As they adopt their stereotypical relative positions they exhibit highly protrusive activity at the lateral plasma membranes. By the early tailbud stage, as cells have initiated elongation, protrusive activity has declined but has become polarized, with the majority of plasma membrane protrusions extending anteriorward. By the mid tailbud stage, elongation is underway and protrusive activity has ceased, with cells appearing almost quiescent. In *sna*>*Bix* embryos very few projections are observed in muscle cells and muscle elongation is impeded.

Using high-speed imaging to visualize non-superficial cells within a developing embryo we have identified filopodial projections present on the lateral surface of muscle cells prior to the initiation, and during the early stages, of tail extension (Movies S8 and S9). The behavior we observed can be divided into three contiguous phases that span muscle elongation (Figure 8). During the initial phase (1), at the beginning of muscle cell elongation at the neurula stage, cells exhibit a highly protrusive activity, with multiple randomly oriented plasma membrane projections. In the next phase (2), during the early stages of tail extension, protrusive activity at the plasma membrane declines but becomes polarized such that projections orient anteriorly. In the final phase (3), once tail extension is underway, the surface of muscle cells becomes largely quiescent, suggesting that intrinsic cellular polarity is established and cell elongation during this period is accomplished in the absence of dynamic protrusive changes at the plasma membrane.

Our observations are intriguing as epithelial cells exhibiting a classical polygonal morphology are believed to achieve intercellular interactions via specialized junctions [29] and have not been reported to exhibit extensive protrusive activity; thus the dynamic protrusive activity that we are observing is novel. On the other hand, it is also likely that this type of spatiotemporal resolution has not been achieved previously, as the high-speed, high-resolution live imaging approach we have undertaken has yet to be applied to *in situ* investigations of cell surface dynamics of epithelial cells within a living organism. This suggests that advances in optical imaging modalities coupled with the availability of subcellularly-localized fluorescent protein reporters exhibiting high signal-to-noise ratios may reveal behaviors not previously documented *in vivo*.

Recent work carried out in living *Xenopus* embryos using a similar approach has shown that, during somitogenesis, presomitic mesoderm cells undergo an increase in the number of filopodial projections as they change their orientation with respect to the notochord [30]. Similarly to our observations of filopodia in the muscle cells of *Ciona*, these protrusions are polarized, in this case being oriented posteriorly towards the nascent somitic boundary [30]. This suggests that the dynamic protrusive activity that we are observing in the developing *Ciona* muscle might be part of an evolutionarily conserved set of cell behaviors that accompany mesoderm formation independent of segmentation events.

The fact that the filopodia are observed only in muscle cells that are undergoing active spatial rearrangements suggests that they may be mediating the interactions occurring among neighboring muscle cells as well as between the muscle and its adjacent tissues during early morphogenesis. Such interactions could involve biomechanical stimulation of cells and/or activation of signal transduction pathways.

A possible role for signal transduction facilitated by muscle cell filopodia is consistent with findings in both *Ciona* and *Xenopus* that mutations in planar cell polarity genes that inhibit the convergent extension of mesodermal cells also affect the protrusive activity of these cells [24], [31]. A link between cell surface signaling and tissue elongation is also supported by studies in *C. elegans*, where mutations of the Rho kinase gene *let*-*502* inhibit myosin-mediated cell elongation [32].

The cell surface projections that we have described may be required for muscle cell elongation, given that inhibition of their formation is associated with defects in muscle patterning in both *sna*>*Bix* and cytochalasin D treated embryos. Future work will focus on a detailed characterization of the molecular architecture of *Ciona* muscle cell filopodia and targeted analysis of their role in muscle cell morphogenesis. Preliminary studies carried out using mutant versions of *C. intestinalis* Rho GTPases [33] and WASP [34], [35] suggest that these candidate factors may not play a direct role in filopodia formation (data not shown).

Given the unilateral extension of the muscle, it will also be important in the future to establish whether patterned protein localization is observed within these muscle cells, as has been documented in epithelia during germband extension in *Drosophila* [29].

### The role of muscle cell elongation in tail extension

Having defined the behaviors of muscle cells operating in a wild type embryo, we sought to examine them in a developmentally perturbed context. Using the *sna*>*Bix* construct to perturb muscle formation, we measured the effects of this perturbation on muscle cell elongation. Bix1 is a homeodomain protein that likely acts as a transcriptional repressor [21], and that has previously been shown to impair tail extension when expressed in developing *Ciona* muscle cells [22]. In wild type embryos, muscle cells appear to be actively driving tail extension in cooperation with notochord cells, through their coordinate elongation, while in muscle cells expressing Bix1, muscle elongation is impaired, thus constraining tail extension [22]. These results are consistent with findings from studies in other ascidian species. Tail morphogenesis in the absence of muscle cells has been described in ascidian embryos produced from interspecific hybridization of the urodele *Molgula oculata* and the anural *M. occulta*, where only short tails were formed through convergent extension of the notochord alone [36]. Likewise, Munro and Odell [37] observed no tail extension in embryos of the urodele ascidian *Boltenia villosa* lacking muscle and neural tissue.

Our results demonstrate that tail extension in *Ciona* is driven not only by the axially positioned notochord, but also by the paraxially positioned muscle. Given the intimate association and coordinated elongation of notochord and muscle, it is likely that some level of intercellular communication exists between these two developing tissues. It will therefore be important to apply the suite of tools and methodologies that we have developed to the investigation of notochord cell behavior. It will also be of interest to determine what interactions exist between the notochord and the muscle cells, and to quantify the respective contributions of these tissues to the morphogenesis of the ascidian tail. Such studies will provide a detailed model for understanding the cell behaviors and interactions underlying tail extension, a process that is common to all chordate lineages.

In conclusion, our work exploits high resolution *in vivo* imaging to reveal the cellular behaviors underlying tail extension and establishes muscle development in *Ciona* as an *in vivo* model for analyzing how changes in cell geometry affect morphogenesis of a tissue. Moreover, muscle cell development in *Ciona* mirrors many aspects of muscle cell gene regulation and morphogenesis documented in other organisms, and as such may serve as a paradigm for related studies of *in vivo* cellular behaviors. The high-resolution multi-spectral live imaging approach we have exploited provides the first multi-dimensional blueprint of muscle cell behavior operating *in situ* during normal development, and shows that *in vivo* imaging of genetically-encoded fluorescent proteins enables direct quantitative comparisons of cell behaviors in wild type and perturbed embryos.

## Supporting Information

Figure S1 Synchronous development of imaged and control embryos. (A) Embryo imaged by laser scanning for 2 hours during tail extension. The embryo was maintained at ambient room temperature of 21°C and the photomicrograph was captured at 10 hours post fertilization. (B, C) Wild type embryos from the same fertilization as the embryo in (A). Wild type embryos were cultures in parallel with the imaged embryo. Control embryos were maintained under conditions identical to those of the imaged embryos, excluding exposure to laser excitation. Imaged and control embryos were synchronous as measured by rate of tail extension, time to otolith melanization, and onset of muscle contractions.
(9.16 MB TIF)

Figure S2 Tail extension is perturbed in embryos electroporated with *sna*>*Bix*. (A) Brightfield images of wild type and *sna*>*Bix* embryos at mid tailbud and late tailbud stages. Time stamps are shown in each image. (B) Change in tail length in wild type and *sna*>*Bix* embryos for the period of development represented in panel (A).
(2.92 MB TIF)

Figure S3 Muscle cells are correctly specified in embryos electroporated with *sna*>*Bix*. Expression of marker genes of muscle differentiation in wild type and *sna*>*Bix* embryos. (A, B) Expression of muscle actin in wild type and *sna*>*Bix* embryos. (C, D) Expression of myosin regulatory light chain (MRLC) in wild type and *sna*>*Bix* embryos. (E, F) Expression of troponin I in wild type and *sna*>*Bix* embryos. Scale bars, 40 µm.
(5.47 MB TIF)

Figure S4 Phenotypes and movement of wild type and *sna*>*Bix* larvae. A time series is shown with *sna*>*Bix* larvae (left) and wild type larvae (right). Images were taken at two second intervals. Arrows indicate the movement of the two wild type larvae due to muscle contractions during the time series. Note that neither of the *sna*>*Bix* larvae has changed position. Scale bars, 200 µm.
(1.56 MB TIF)

Figure S5 Organization of myofibrils in wild type and *sna*>*Bix* larvae, as visualized by rhodamine phalloidin labeling. (A) Myofibrils in a wild type larva. (B) Myofibrils in a larva electroporated with *sna*>*Bix*. Myofibrils in the *sna*>*Bix* larvae are less numerous and less organized than those in wild type larvae.
(2.39 MB TIF)

Figure S6 Organization of myofibrils in wild type and *sna*>*Bix* muscle cells, as visualized by transmission electron microscopy (TEM). (A) Low magnification TEM image of a semi-longitudinal section of the tail of a *Ciona* larva. Muscle cells are distinguishable based on their shape and on the high number of mitochondria. (B) Higher magnification of the area outlined by a red rectangle in panel A, encompassing the boundary between adjacent muscle cells. On each side of the cells' boundary, numerous myofibrils can be seen. (C) When a ∼30,000× magnification is employed, regularly patterned myofibrils, indicated by arrows, can be distinguished. (D) Low magnification TEM image of the tail of a *Ciona* larva electroporated with *sna*>*Bix*. An irregularly shaped muscle cell is boxed by a red rectangle and shown at a higher magnification in (E). (F) Higher magnification image of the region boxed by a red rectangle in (E). Arrows indicate disorganized myofilaments.
(5.46 MB PDF)

Figure S7 Changes in muscle cell geometry during tail extension visualized with the vital dye FM4-64. (A) Embryo electroporated with *Tbx6b*>*H2B-GFP* to mark muscle cells and incubated with FM4-64 to label cell membranes. (B) Graph of average muscle cell shape (ratio of length to height; n = 5) from the *Tbx6b*>*H2B-GFP*+FM4-64 embryo plotted against tail length, with standard deviation shown for each data point. (C) Embryo co-electroporated with *sna*>*Bix* to perturb muscle development and *Tbx6b*>*H2B-GFP* to mark muscle cell nuclei, and incubated with FM4-64, which labels cell membranes. (D) Graph of average muscle cell shape (ratio of length to height; n = 5) from the *sna*>*Bix*+*Tbx6b*>*H2B-GFP*+FM4-64 embryo plotted against tail length of a wild type embryo monitored in parallel, with standard deviation shown for each data point. Scale bars, 40 µm.
(2.81 MB TIF)

Movie S1 Time lapse imaging of an embryo electroporated with *sna*>*Venus*.
(5.69 MB MOV)

Movie S2 Time lapse imaging of tail extension is shown in a wild type embryo coelectroporated with *sna*>*GPI-GFP* and *sna*>*H2B-RFP*.
(1.94 MB MOV)

Movie S3 Movement of muscle cell nuclei in a wild type embryo during tail extension. Tracked cells are false-colored.
(0.27 MB MOV)

Movie S4 Wide field time-lapse of late tailbud or larval stage *sna*>*Bix* embryos.
(3.29 MB MOV)

Movie S5 Movement of muscle cell nuclei in a *sna*>*Bix* embryo during tail extension. Tracked cells are false-colored.
(0.34 MB MOV)

Movie S6 Changes in muscle cell shape in a wild type embryo during tail extension.
(0.83 MB MOV)

Movie S7 Changes in muscle cell shape in a *sna*>*Bix* embryo during tail extension.
(0.94 MB MOV)

Movie S8 High speed imaging of a wild type embryo electroporated with *sna*>*GPI-GFP*. Note protrusive activity of the muscle cell surfaces during the neurula and early tailbud stages.
(8.95 MB MOV)

Movie S9 High speed imaging of a wild type embryo electroporated with *Tbx6b*>*PH-YFP*.
(10.34 MB MOV)

Movie S10 High speed imaging of an embryo coelectroporated with *sna*>*Bix* and *Tbx6b*>*PH-YFP*.
(1.00 MB MOV)
