# Supplementary figures and images for: Dynamic and Polarized Muscle Cell Behaviors Accompany Tail Morphogenesis in the Ascidian Ciona intestinalis
Source: PLoS One. 2007 Aug 8;2(8):e714. doi: 10.1371/journal.pone.0000714 (PMC1934933; doi:10.1371/journal.pone.0000714)

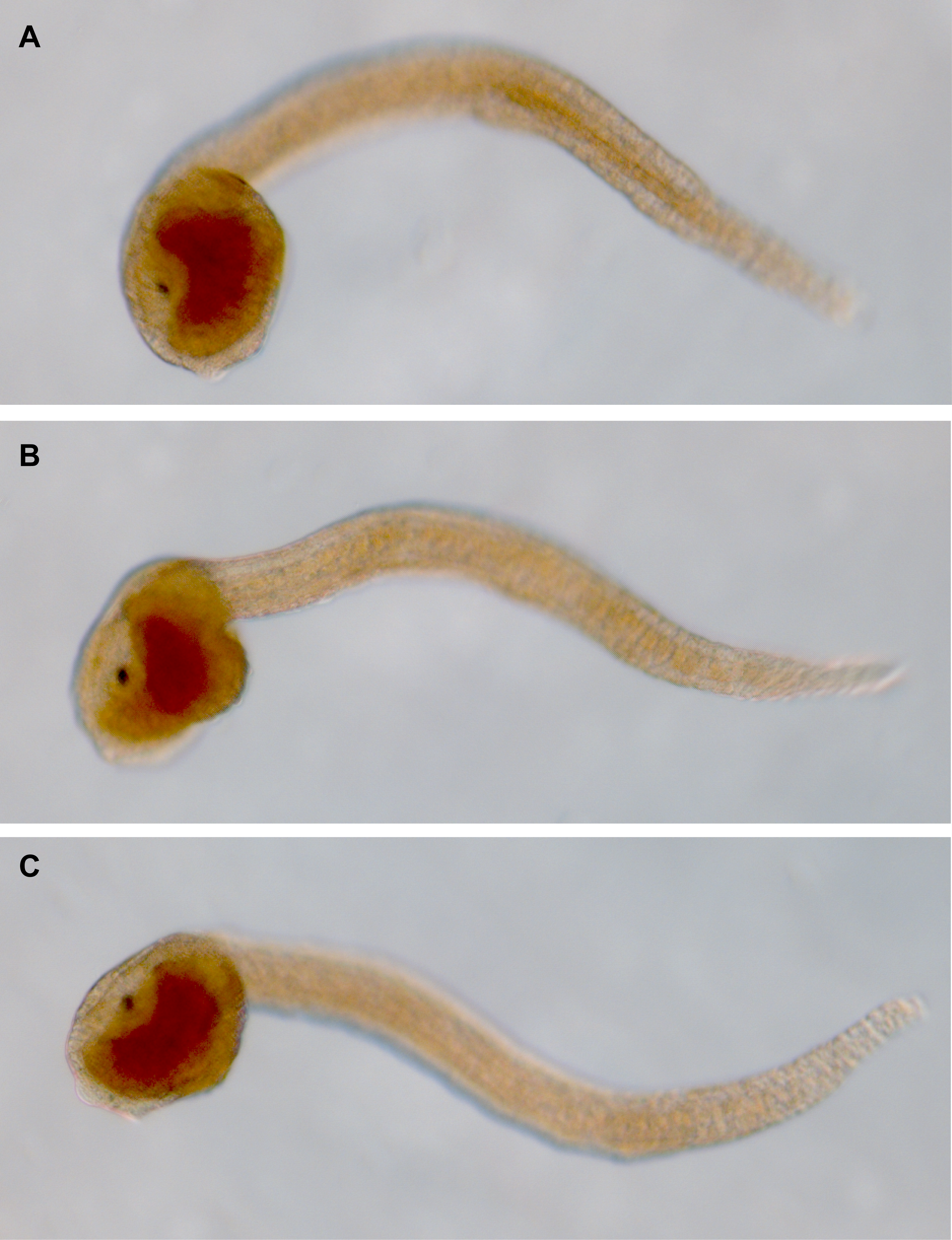

Supplement: Figure S1 — Synchronous development of imaged and control embryos. (A) Embryo imaged by laser scanning for 2 hours during tail extension. The embryo was maintained at ambient room temperature of 21°C and the photomicrograph was captured at 10 hours post fertilization. (B, C) Wild type embryos from the same fertilization as the embryo in (A). Wild type embryos were cultures in parallel with the imaged embryo. Control embryos were maintained under conditions identical to those of the imaged embryos, excluding exposure to laser excitation. Imaged and control embryos were synchronous as measured by rate of tail extension, time to otolith melanization, and onset of muscle contractions. (9.16 MB TIF) [file pone.0000714.s001.tif]

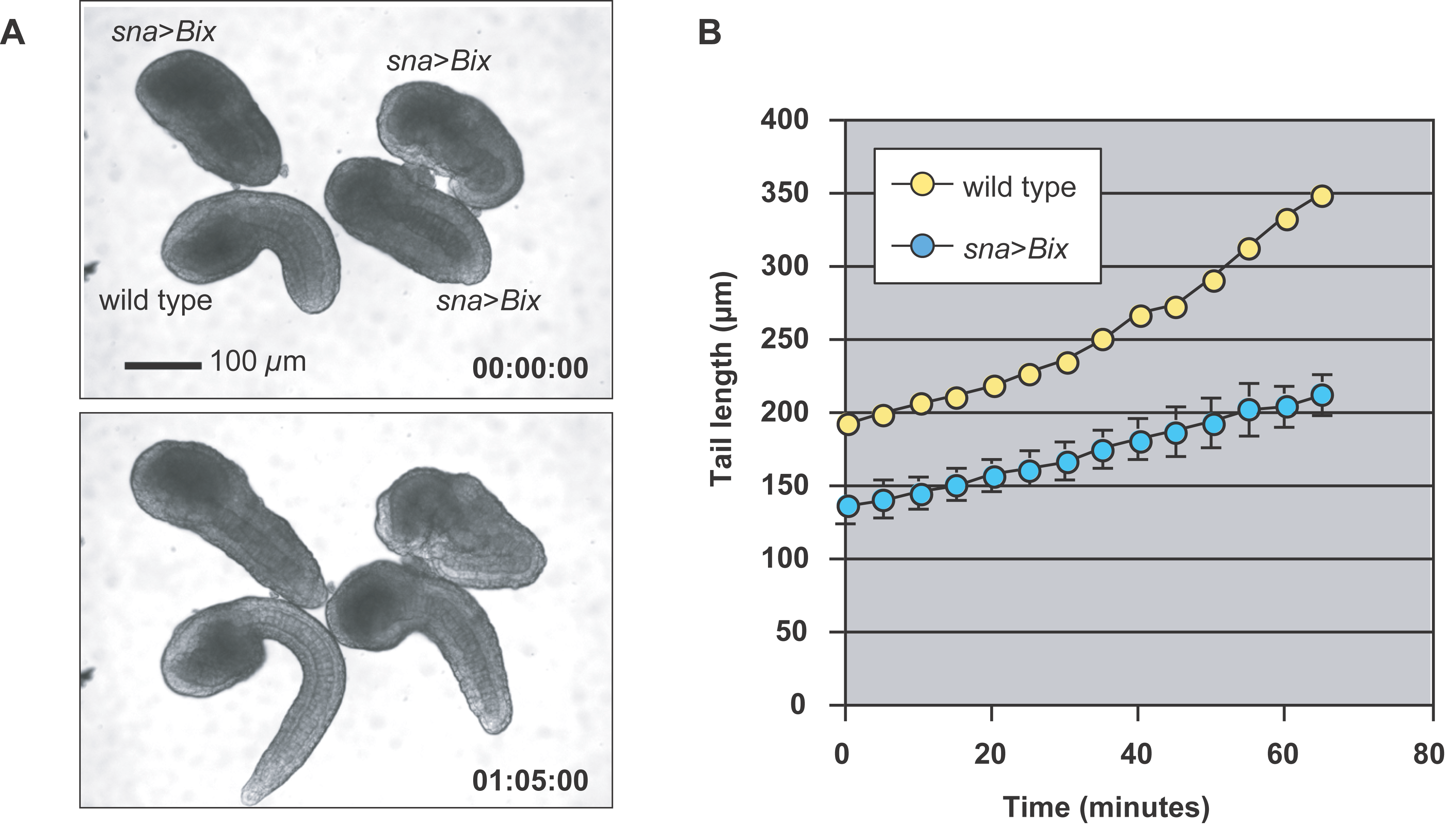

Supplement: Figure S2 — Tail extension is perturbed in embryos electroporated with sna>Bix. (A) Brightfield images of wild type and sna>Bix embryos at mid tailbud and late tailbud stages. Time stamps are shown in each image. (B) Change in tail length in wild type and sna>Bix embryos for the period of development represented in panel (A). (2.92 MB TIF) [file pone.0000714.s002.tif]

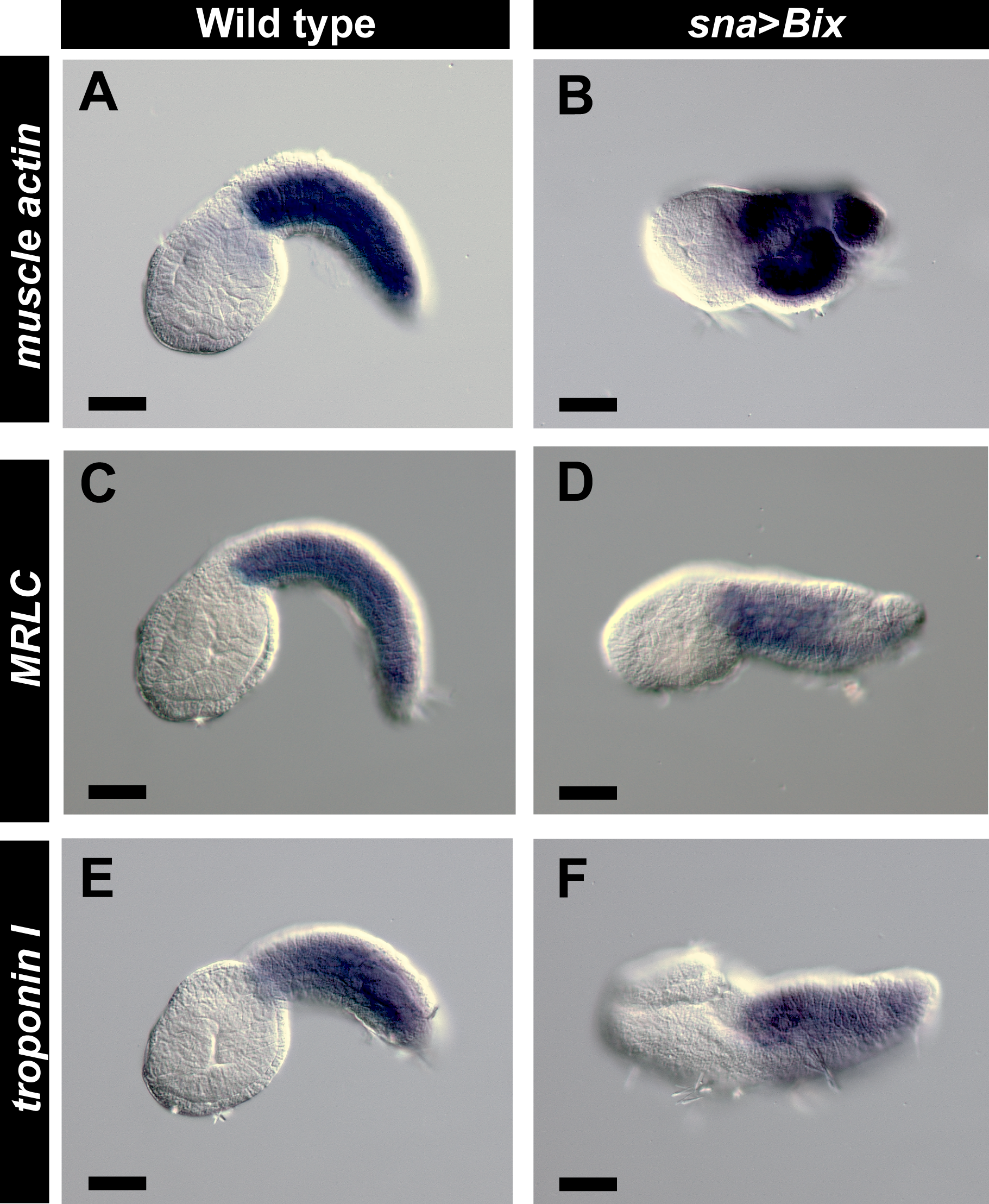

Supplement: Figure S3 — Muscle cells are correctly specified in embryos electroporated with sna>Bix. Expression of marker genes of muscle differentiation in wild type and sna>Bix embryos. (A, B) Expression of muscle actin in wild type and sna>Bix embryos. (C, D) Expression of myosin regulatory light chain (MRLC) in wild type and sna>Bix embryos. (E, F) Expression of troponin I in wild type and sna>Bix embryos. Scale bars, 40 µm. (5.47 MB TIF) [file pone.0000714.s003.tif]

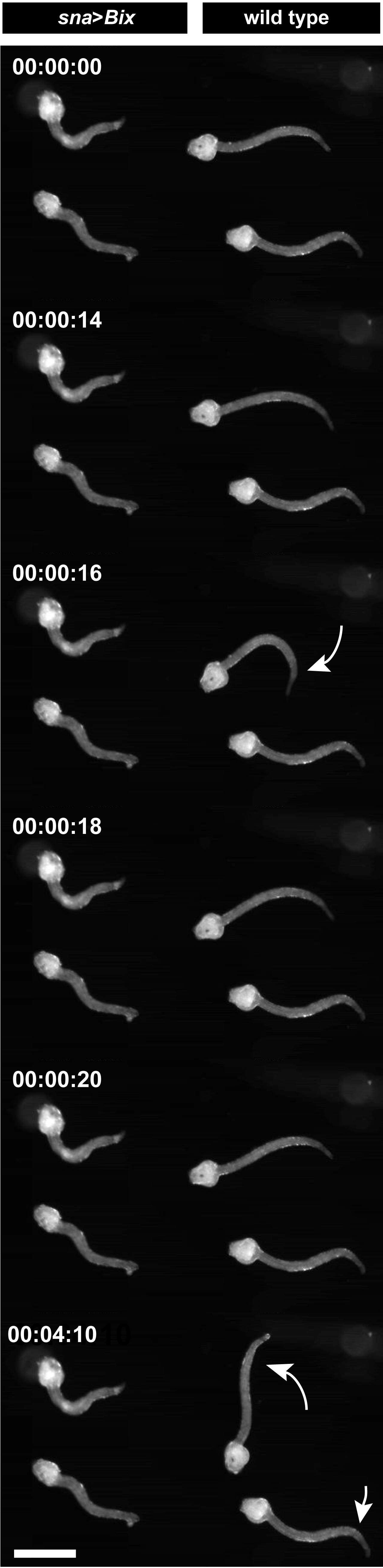

Supplement: Figure S4 — Phenotypes and movement of wild type and sna>Bix larvae. A time series is shown with sna>Bix larvae (left) and wild type larvae (right). Images were taken at two second intervals. Arrows indicate the movement of the two wild type larvae due to muscle contractions during the time series. Note that neither of the sna>Bix larvae has changed position. Scale bars, 200 µm. (1.56 MB TIF) [file pone.0000714.s004.tif]

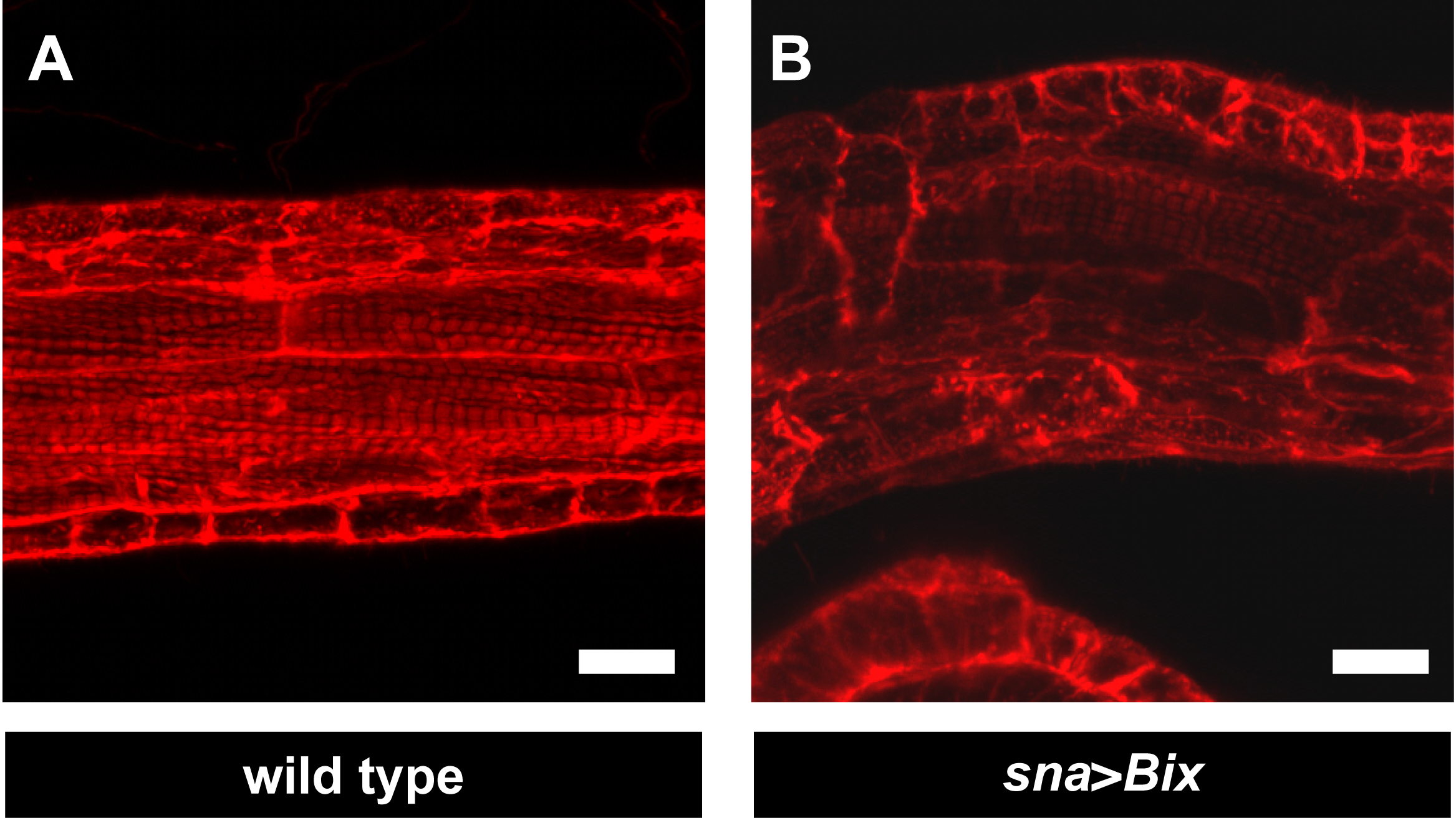

Supplement: Figure S5 — Organization of myofibrils in wild type and sna>Bix larvae, as visualized by rhodamine phalloidin labeling. (A) Myofibrils in a wild type larva. (B) Myofibrils in a larva electroporated with sna>Bix. Myofibrils in the sna>Bix larvae are less numerous and less organized than those in wild type larvae. (2.39 MB TIF) [file pone.0000714.s005.tif]

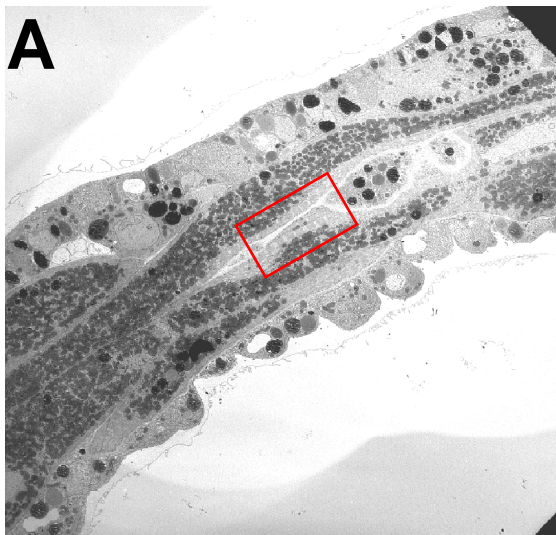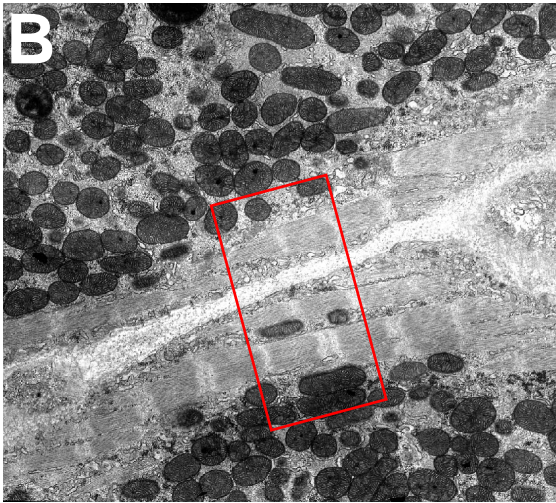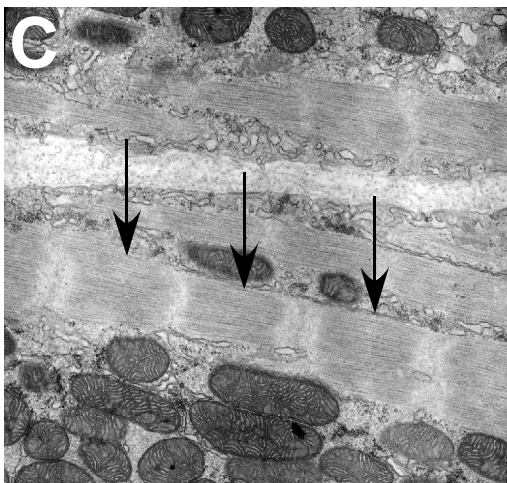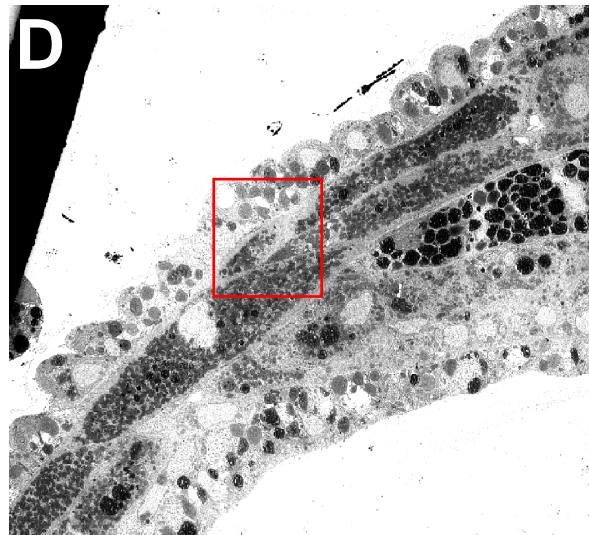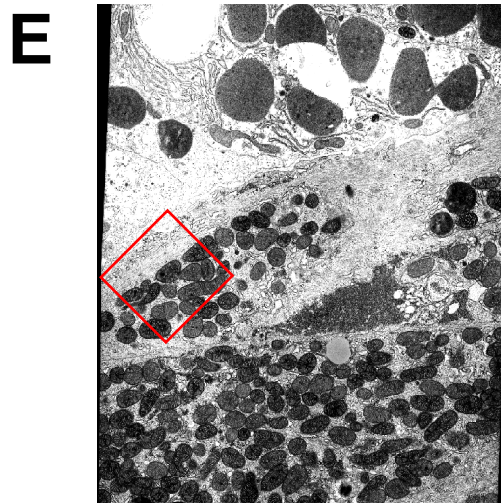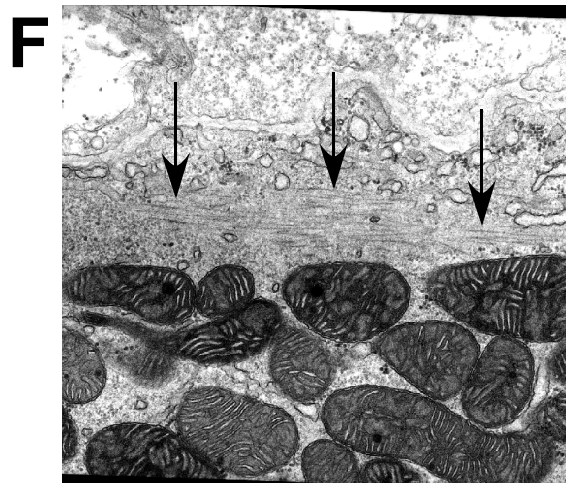

Supplement: Figure S6 — Organization of myofibrils in wild type and sna>Bix muscle cells, as visualized by transmission electron microscopy (TEM). (A) Low magnification TEM image of a semi-longitudinal section of the tail of a Ciona larva. Muscle cells are distinguishable based on their shape and on the high number of mitochondria. (B) Higher magnification of the area outlined by a red rectangle in panel A, encompassing the boundary between adjacent muscle cells. On each side of the cells' boundary, numerous myofibrils can be seen. (C) When a ∼30,000× magnification is employed, regularly patterned myofibrils, indicated by arrows, can be distinguished. (D) Low magnification TEM image of the tail of a Ciona larva electroporated with sna>Bix. An irregularly shaped muscle cell is boxed by a red rectangle and shown at a higher magnification in (E). (F) Higher magnification image of the region boxed by a red rectangle in (E). Arrows indicate disorganized myofilaments. (5.46 MB PDF) [file pone.0000714.s006.pdf]

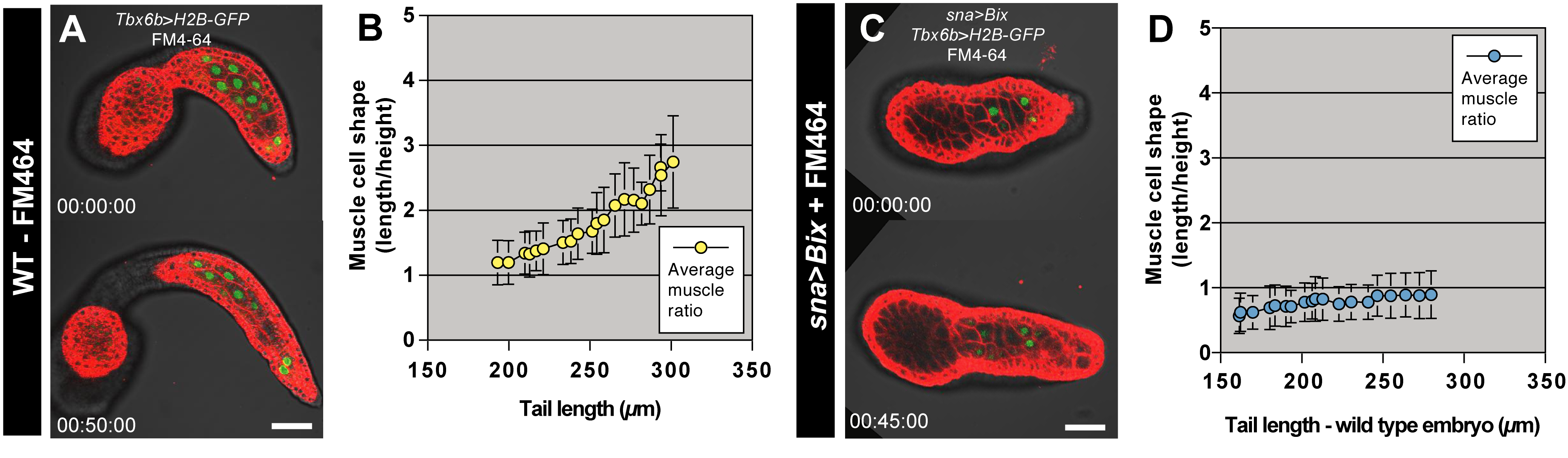

Supplement: Figure S7 — Changes in muscle cell geometry during tail extension visualized with the vital dye FM4-64. (A) Embryo electroporated with Tbx6b>H2B-GFP to mark muscle cells and incubated with FM4-64 to label cell membranes. (B) Graph of average muscle cell shape (ratio of length to height; n = 5) from the Tbx6b>H2B-GFP+FM4-64 embryo plotted against tail length, with standard deviation shown for each data point. (C) Embryo co-electroporated with sna>Bix to perturb muscle development and Tbx6b>H2B-GFP to mark muscle cell nuclei, and incubated with FM4-64, which labels cell membranes. (D) Graph of average muscle cell shape (ratio of length to height; n = 5) from the sna>Bix+Tbx6b>H2B-GFP+FM4-64 embryo plotted against tail length of a wild type embryo monitored in parallel, with standard deviation shown for each data point. Scale bars, 40 µm. (2.81 MB TIF) [file pone.0000714.s007.tif]
